# Supplementary figures and images for: Long-term continuously monocropped peanut significantly changed the abundance and composition of soil bacterial communities
Source: PeerJ. 2020 Apr 28;8:e9024. doi: 10.7717/peerj.9024 (PMC7194089; doi:10.7717/peerj.9024)

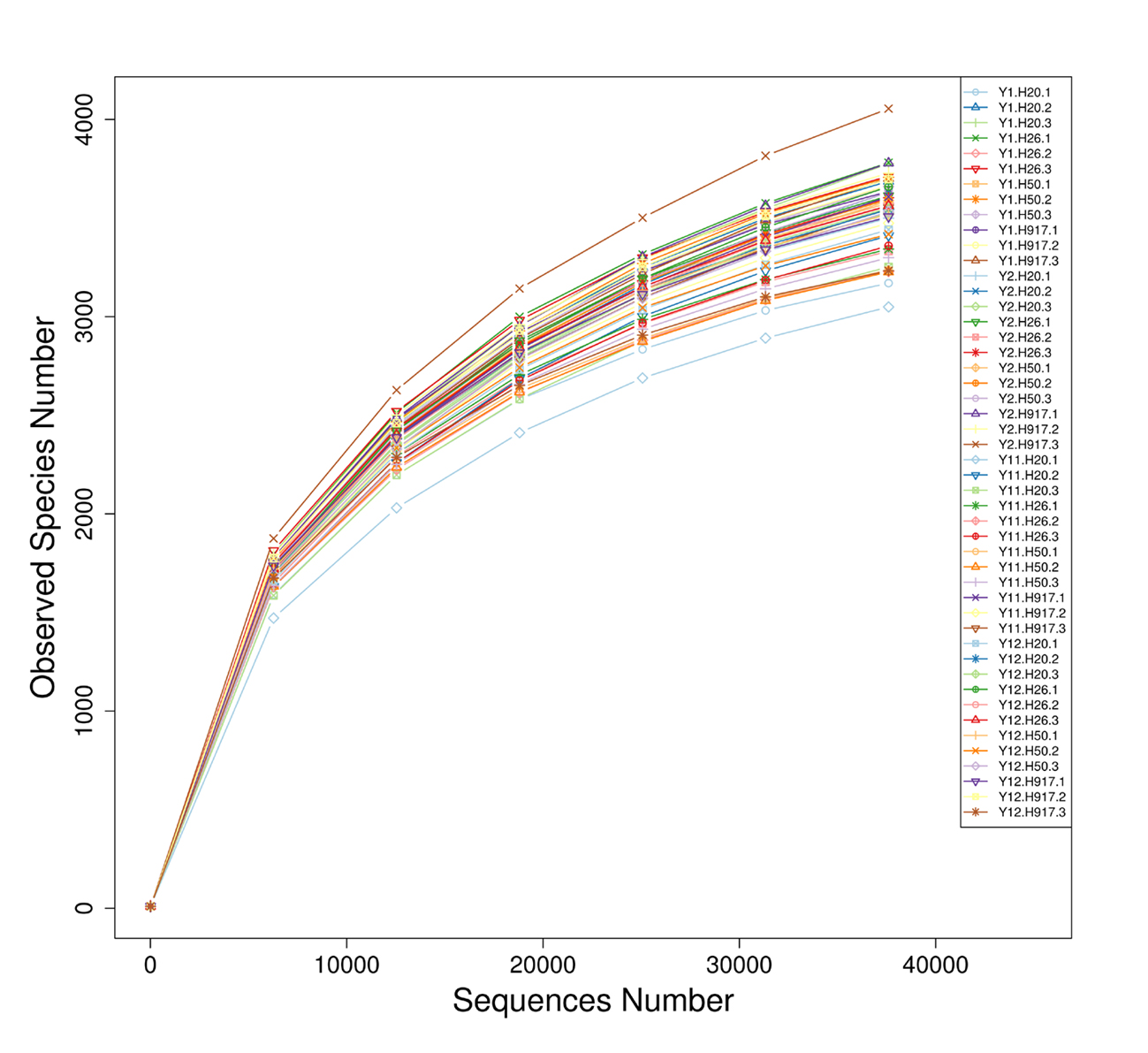

Supplement: Figure S1 [file peerj-08-9024-s001.jpg]

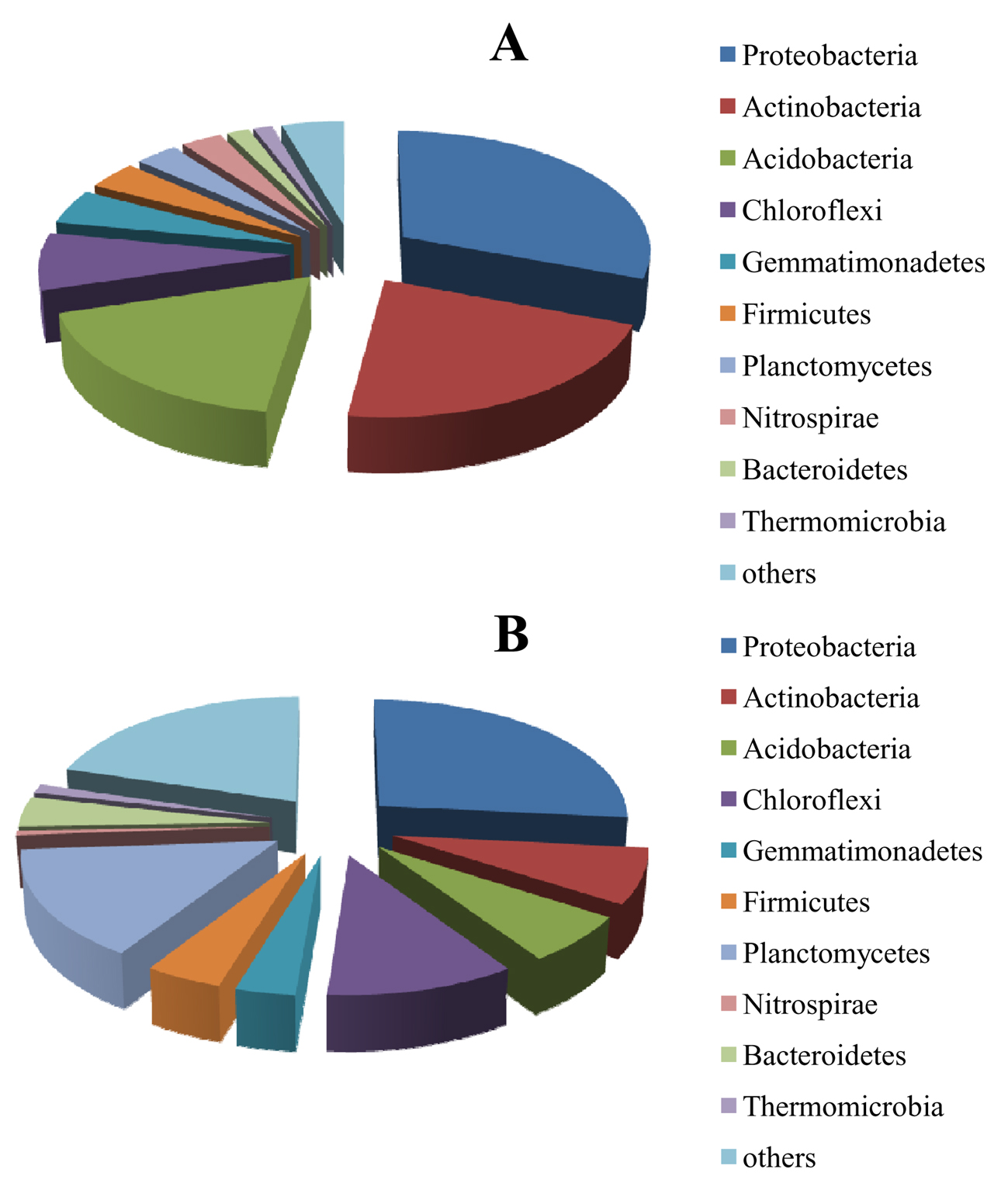

Supplement: Figure S2 [file peerj-08-9024-s002.jpg]

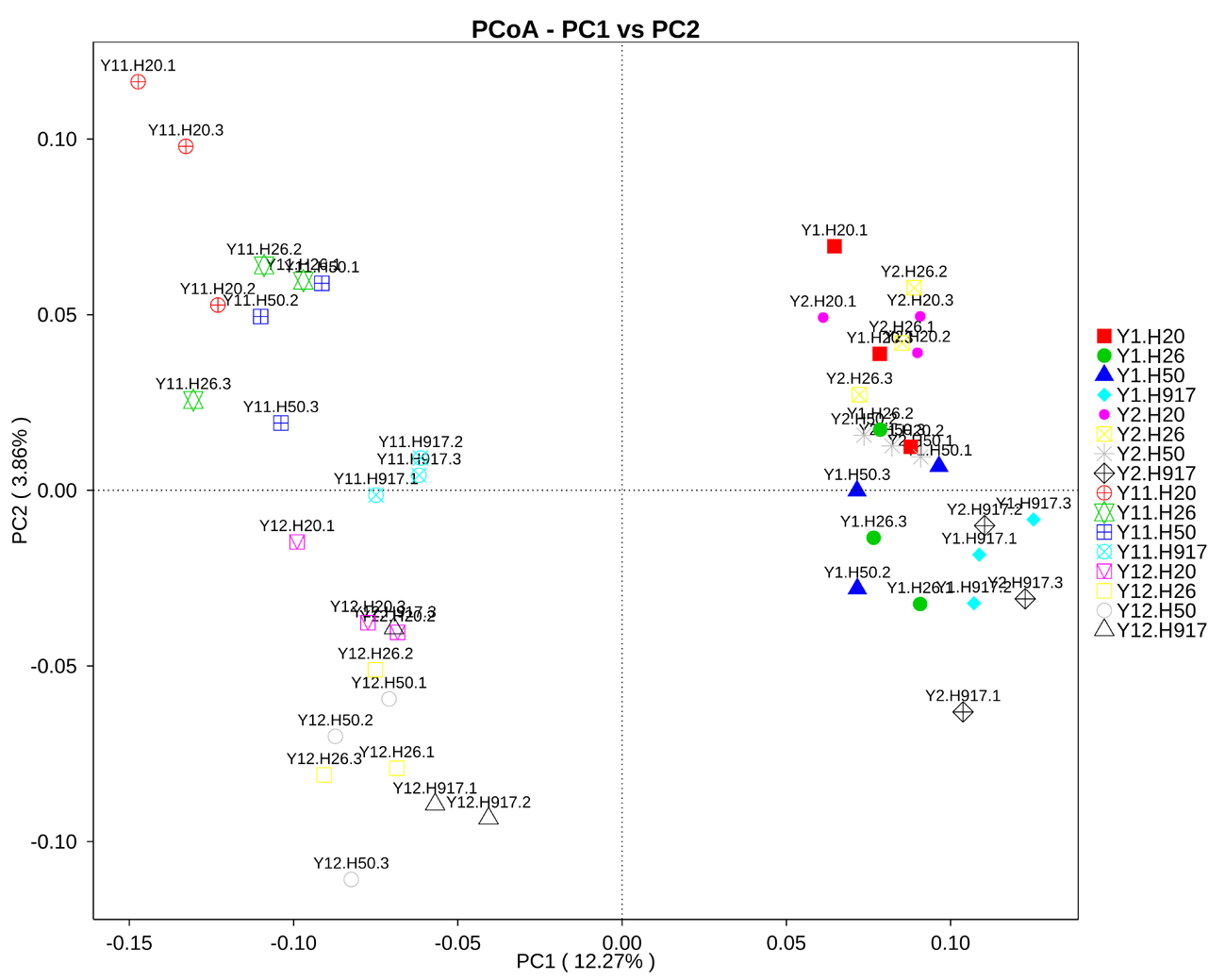

Supplement: Figure S3 [file peerj-08-9024-s003.jpg]

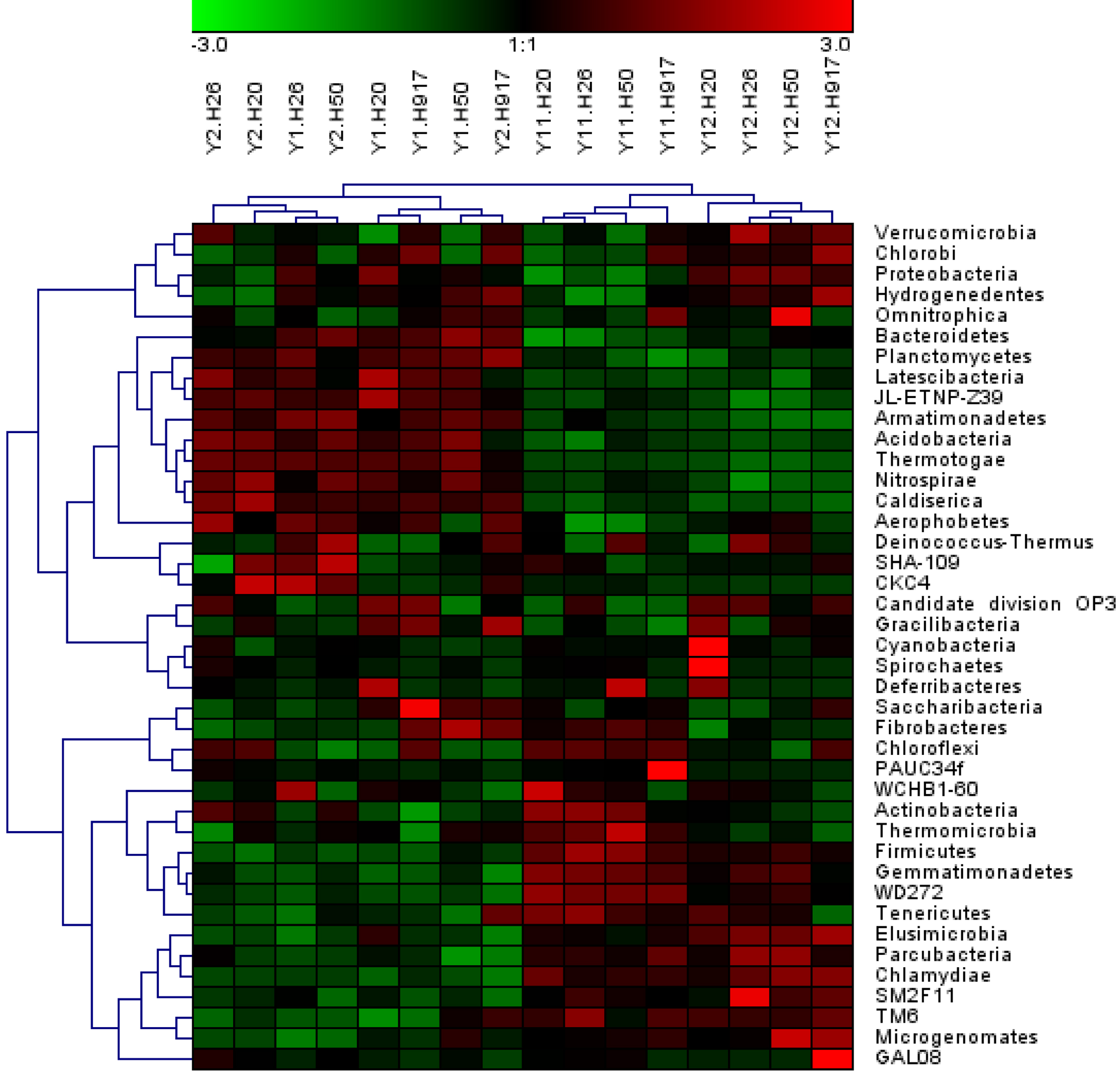

Supplement: Figure S4 [file peerj-08-9024-s004.jpg]

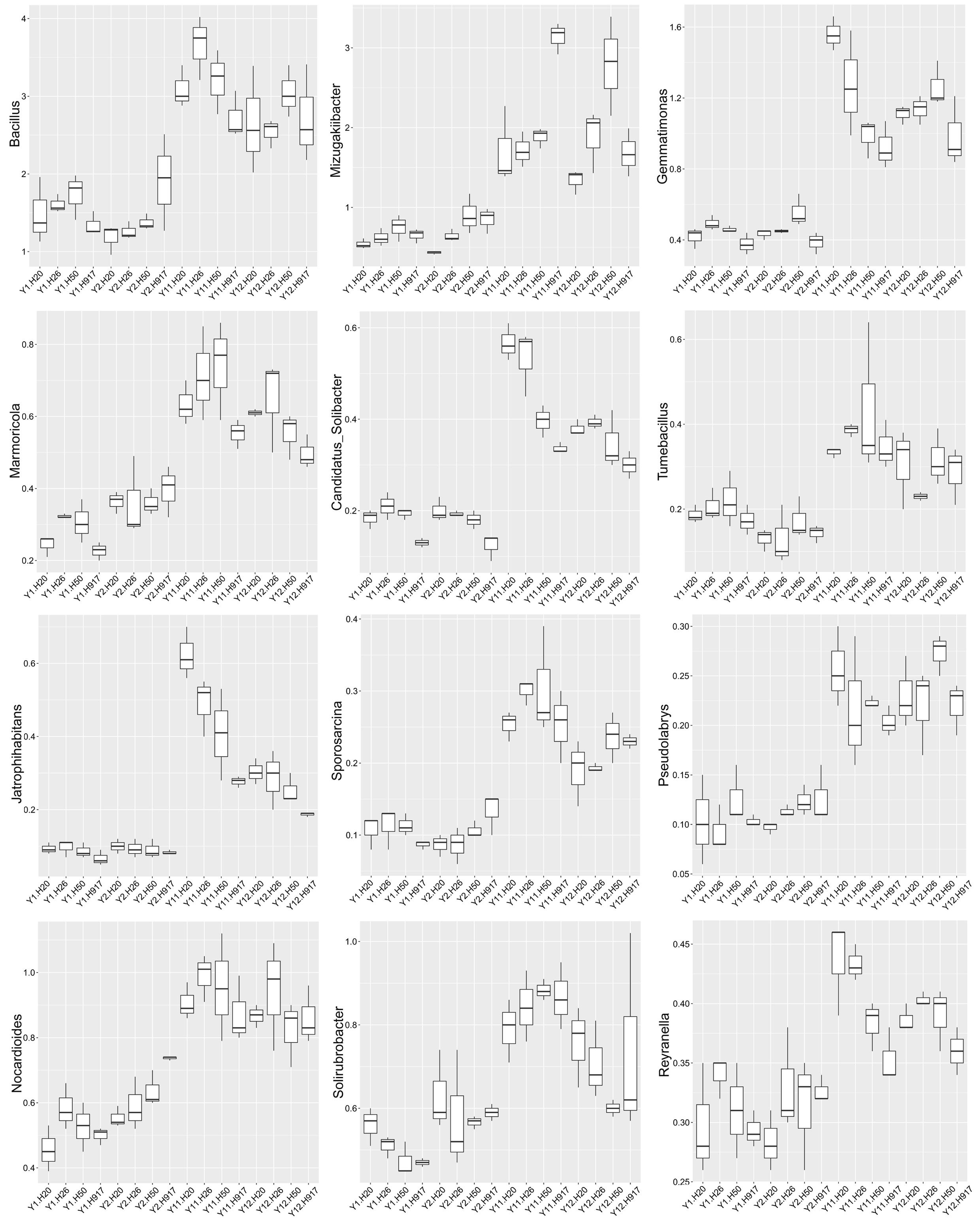

Supplement: Figure S5 [file peerj-08-9024-s005.jpg]

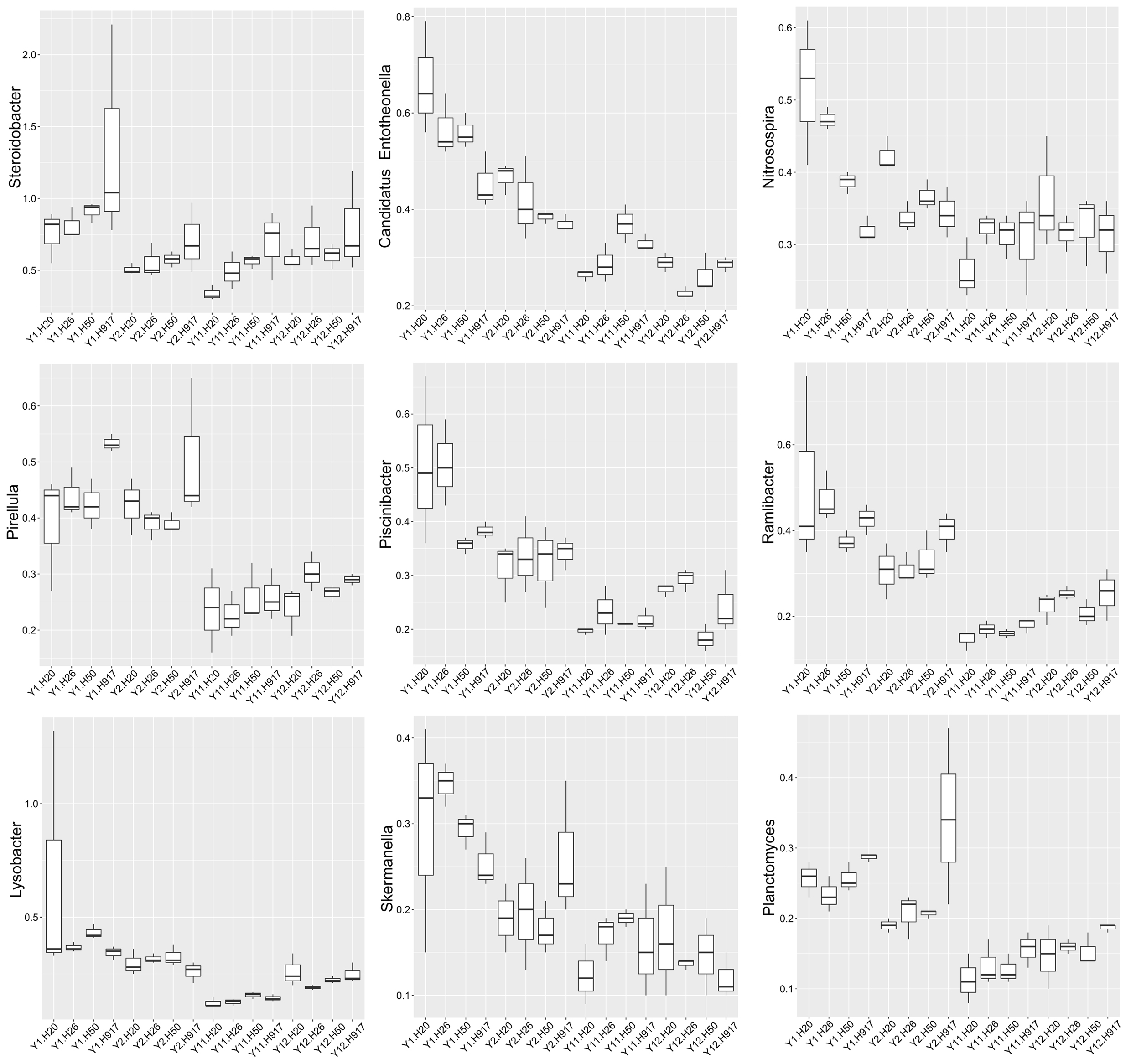

Supplement: Figure S6 [file peerj-08-9024-s006.jpg]

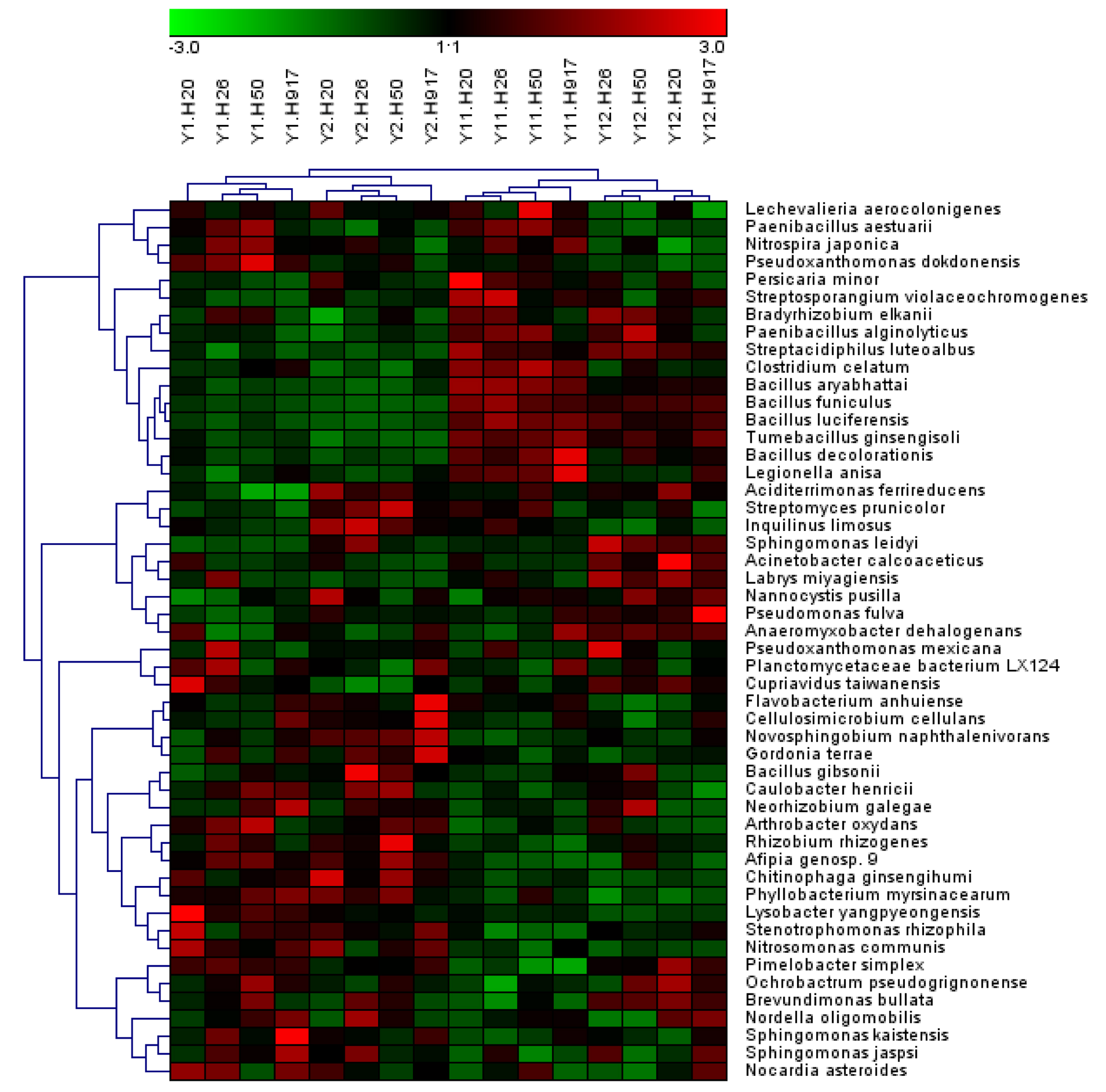

Supplement: Figure S7 [file peerj-08-9024-s007.jpg]

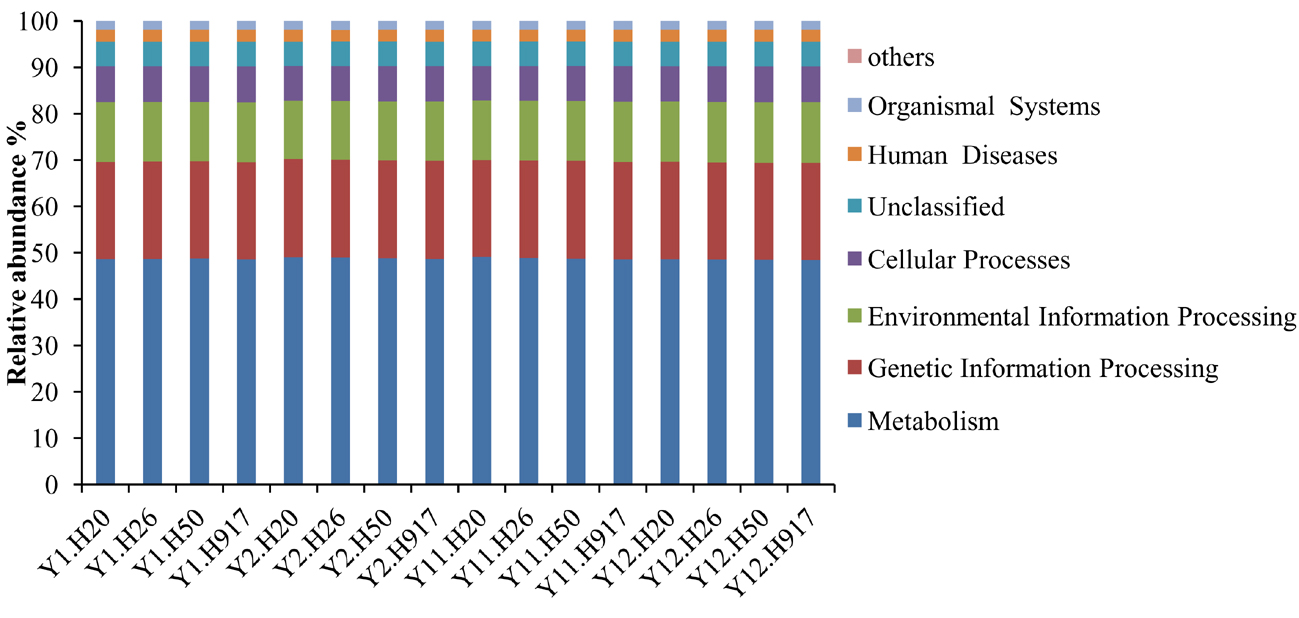

Supplement: Figure S8 [file peerj-08-9024-s008.jpg]

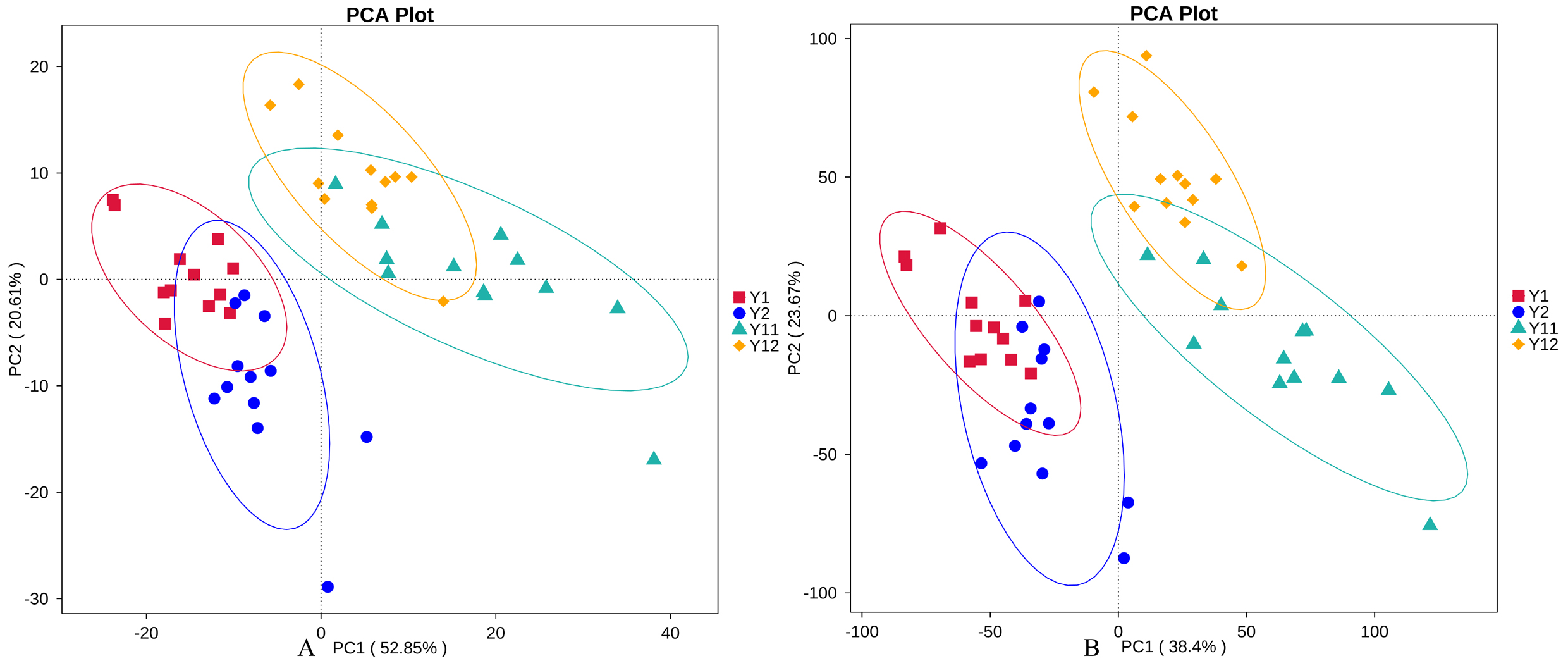

Supplement: Figure S9 [file peerj-08-9024-s009.jpg]
