# Supplementary material for: Long-term continuously monocropped peanut significantly changed the abundance and composition of soil bacterial communities
Source: PeerJ. 2020 Apr 28;8:e9024. doi: 10.7717/peerj.9024 (PMC7194089; doi:10.7717/peerj.9024)
Supplement: Supplemental Information 11 [file peerj-08-9024-s011.docx]

Table S1. Soil physiochemical properties in plots that were monocropped with peanuts for different periods

|  | pH | organic matter  g kg^−1^ | avilable N  mg kg^−1^ | avilable P  mg kg^−1^ | avilable K mg kg^−1^ |
| --- | --- | --- | --- | --- | --- |
| Y1.H20 | 7.20±0.02 | 10.26±0.11 | 77.37±1.61 | 41.33±1.90 | 103.37±0.73 |
| Y1.H26 | 7.77±0.08 | 10.37±0.61 | 78.74±1.28 | 41.65±1.41 | 104.31±0.58 |
| Y1.H50 | 7.93±0.03 | 9.74±0.43 | 69.77±0.75 | 44.49±1.40 | 111.53±1.54 |
| Y1.H917 | 8.05±0.05 | 6.59±0.65 | 88.2±2.65 | 40.15±2.66 | 107.87±0.67 |
| Y2.H20 | 8.37±0.03 | 9.87±0.57 | 75.2±1.17 | 39.37±0.34 | 99.42±0.76 |
| Y2.H26 | 8.56±0.02 | 9.25±0.67 | 86.38±0.55 | 48.58±0.41 | 120.67±1.25 |
| Y2.H50 | 8.30±0.03 | 7.03±0.35 | 77.56±2.42 | 45.21±2.12 | 112.17±1.31 |
| Y2.H917 | 8.59±0.04 | 15.53±0.10 | 94.96±1.51 | 40.33±0.31 | 102.3±1.32 |
| Y11.H20 | 6.72±0.02 | 11.32±0.42 | 70.34±0.37 | 17.02±0.75 | 68.15±0.76 |
| Y11.H26 | 6.46±0.03 | 12.12±0.21 | 76.7±2.90 | 19.52±0.97 | 78.12±0.46 |
| Y11.H50 | 6.63±0.04 | 8.74±0.31 | 74.63±3.70 | 15.12±0.75 | 60.11±0.81 |
| Y11.H917 | 6.64±0.06 | 10.9±0.35 | 80.92±2.66 | 17.26±1.51 | 79.04±0.60 |
| Y12.H20 | 6.92±0.03 | 10.36±0.40 | 88.99±2.15 | 15.12±1.15 | 59.98±1.10 |
| Y12.H26 | 6.82±0.03 | 11.01±0.17 | 87.97±3.21 | 20.18±0.85 | 80.85±0.87 |
| Y12.H50 | 6.55±0.04 | 10.23±0.35 | 77.33±3.08 | 18.72±0.17 | 86.13±0.83 |
| Y12.H917 | 6.72±0.02 | 10.22±0.21 | 85.2±2.16 | 21.12±0.14 | 77.03±0.83 |

|  | Y1.20 | Y1.26 | Y1.50 | Y1.917 | Y2.20 | Y2.26 | Y2.50 | Y2.917 | Y11.20 | Y11.26 | Y11.50 | Y11.917 | Y12.20 | Y12.26 | Y12.50 | Y12.917 |
| --- | --- | --- | --- | --- | --- | --- | --- | --- | --- | --- | --- | --- | --- | --- | --- | --- |
| Acidobacteria | 471±16 | 468±8 | 460±5 | 464±6 | 463±9 | 454±3 | 455±7 | 450±3 | 426±9 | 421±7 | 433±5 | 438±6 | 438±10 | 434±10 | 417±2 | 437±8 |
| Planctomycetes | 712±87 | 756±41 | 736±21 | 739±26 | 730±24 | 753±72 | 718±25 | 783±72 | 570±43 | 609±24 | 601±14 | 631±13 | 625±42 | 653±7 | 604±21 | 620±18 |
| Nitrospirae | 50±1 | 54±2 | 49±2 | 51±3 | 49±2 | 52±2 | 50±1 | 49±2 | 44±3 | 43±5 | 42±2 | 45±1 | 47±3 | 44±4 | 41±1 | 43±3 |
| Bacteroidetes | 201±15 | 212±3 | 223±11 | 205±4 | 175±2 | 170±14 | 206±6 | 222±14 | 148±11 | 157±12 | 154±2 | 187±2 | 187±13 | 194±5 | 211±5 | 210±12 |
| Armatimonadetes | 157±8 | 179±1 | 158±10 | 162±2 | 167±7 | 161±6 | 168±11 | 169±6 | 145±2 | 141±3 | 137±2 | 150±3 | 120±9 | 115±7 | 110±9 | 111±18 |
| Latescibacteria | 40±2 | 40±1 | 39±2 | 41±2 | 40±2 | 42±1 | 43±3 | 40±1 | 32±2 | 27±2 | 30±3 | 31±3 | 32±2 | 32±1 | 33±1 | 37±6 |
| JL-ETNP-Z39 | 7±2 | 6±1 | 6±1 | 5±1 | 7±1 | 5±1 | 5±1 | 6±1 | 4±1 | 5±1 | 4±1 | 5±1 | 5±1 | 6±1 | 4±1 | 6±1 |
| Thermotogae | 6±1 | 8±1 | 7±1 | 8±2 | 7±1 | 7±1 | 8±1 | 6±1 | 4±1 | 4±1 | 4±1 | 5±1 | 4±1 | 5±1 | 4±1 | 5±1 |
| Caldiserica | 1±0 | 1±0 | 1±0 | 1±0 | 1±0 | 1±0 | 1±0 | 1±0 | 1±1 | 1±1 | 1±1 | 1±0 | 1±1 | 1±0 | 1±0 | 1±1 |
| Gemmatimonadetes | 218±14 | 221±5 | 227±4 | 215±9 | 221±8 | 228±12 | 227±8 | 238±3 | 195±8 | 204±4 | 193±6 | 227±2 | 215±6 | 231±3 | 218±4 | 219±18 |
| Firmicutes | 190±19 | 209±4 | 205±5 | 193±10 | 175±8 | 165±12 | 188±6 | 208±15 | 220±2 | 228±4 | 215±10 | 219±4 | 212±12 | 212±8 | 209±3 | 207±3 |
| Elusimicrobia | 58±2 | 46±2 | 60±2 | 55±4 | 44±3 | 43±5 | 50±3 | 49±3 | 50±6 | 45±7 | 50±4 | 51±1 | 56±7 | 53±1 | 52±3 | 56±2 |
| Chlamydiae | 6±1 | 8±3 | 7±1 | 9±3 | 8±2 | 4±2 | 7±2 | 8±2 | 14±2 | 11±3 | 14±4 | 12±1 | 15±2 | 14±3 | 14±2 | 15±3 |
| Parcubacteria | 16±2 | 13±1 | 9±3 | 18±3 | 12±1 | 15±2 | 16±2 | 13±4 | 25±3 | 21±3 | 17±4 | 26±3 | 21±2 | 33±2 | 36±5 | 27±2 |

Table S2. The OTU number of significantly changed taxa at phylum level in plots that were monocropped with peanuts for different periods of time.Table S3. Relative abundance of significantly changed KEGG pathways at KEGG level 2 in plots that were monocropped with peanuts for different periods. Italic type represents the pathways with increased abundance and standard type represents pathways with decreased abundance.

|  | Y1.H20 | Y1.H26 | Y1.H50 | Y1.H917 | Y2.H20 | Y2.H26 | Y2.H50 | Y2.H917 | Y11.H20 | Y11.H26 | Y11.H50 | Y11.H917 | Y12.H20 | Y12.H26 | Y12.H50 | Y12.H917 |
| --- | --- | --- | --- | --- | --- | --- | --- | --- | --- | --- | --- | --- | --- | --- | --- | --- |
| Carbohydrate metabolism | 10.77±0.02 | 10.78±0.02 | 10.80±0.02 | 10.75±0.02 | 10.86±0.04 | 10.87±0.02 | 10.81±0.03 | 10.78±0.02 | 10.63±0.02 | 10.64±0.02 | 10.64±0.01 | 10.60±0.02 | 10.62±0.03 | 10.57±0.02 | 10.54±0.03 | 10.57±0.04 |
| Endocrine system | 0.89±0.00 | 0.88±0.00 | 0.88±0.00 | 0.87±0.01 | 0.89±0.01 | 0.90±0.01 | 0.89±0.00 | 0.88±0.00 | 0.87±0.00 | 0.88±0.00 | 0.87±0.00 | 0.86±0.01 | 0.87±0.00 | 0.86±0.01 | 0.85±0.01 | 0.86±0.01 |
| Excretory system | 0.06±0.00 | 0.05±0.00 | 0.05±0.00 | 0.05±0.00 | 0.05±0.00 | 0.05±0.00 | 0.05±0.00 | 0.05±0.00 | 0.05±0.00 | 0.05±0.00 | 0.05±0.00 | 0.05±0.00 | 0.05±0.00 | 0.05±0.00 | 0.05±0.00 | 0.048±0.00 |
| Nucleotide metabolism | 3.10±0.01 | 3.10±0.01 | 3.11±0.01 | 3.14±0.01 | 3.12±0.05 | 3.08±0.03 | 3.09±0.01 | 3.10±0.01 | 3.01±0.01 | 2.99±0.03 | 3.03±0.01 | 3.06±0.02 | 3.05±0.01 | 3.05±0.02 | 3.07±0.01 | 3.07±0.04 |
| Transport and catabolism | 2.38±0.00 | 2.38±0.01 | 2.39±0.01 | 2.41±0.00 | 2.38±0.01 | 2.37±0.02 | 2.37±0.01 | 2.37±0.00 | 2.31±0.01 | 2.30±0.02 | 2.32±0.01 | 2.34±0.01 | 2.33±0.00 | 2.33±0.02 | 2.34±0.01 | 2.34±0.02 |
| Transcription | 1.79±0.01 | 1.79±0.01 | 1.79±0.01 | 1.80±0.00 | 1.80±0.02 | 1.78±0.01 | 1.78±0.01 | 1.79±0.00 | 1.73±0.00 | 1.73±0.01 | 1.75±0.01 | 1.76±0.00 | 1.76±0.00 | 1.76±0.01 | 1.76±0.00 | 1.77±0.00 |
| Biosynthesis of other secondary metabolites | 1.51±0.00 | 1.51±0.00 | 1.52±0.01 | 1.52±0.00 | 1.52±0.00 | 1.52±0.01 | 1.52±0.01 | 1.51±0.01 | 1.48±0.00 | 1.47±0.00 | 1.49±0.01 | 1.49±0.00 | 1.48±0.00 | 1.48±0.00 | 1.49±0.01 | 1.48±0.01 |
| Aging | 0.43±0.00 | 0.43±0.00 | 0.43±0.01 | 0.43±0.00 | 0.43±0.00 | 0.43±0.00 | 0.43±0.00 | 0.43±0.00 | 0.43±0.00 | 0.43±0.00 | 0.43±0.00 | 0.43±0.00 | 0.43±0.00 | 0.43±0.00 | 0.43±0.00 | 0.43±0.00 |
| *Membrane transport* | 9.05±0.08 | 8.99±0.06 | 8.92±0.02 | 8.99±0.09 | 8.80±0.09 | 8.84±0.06 | 8.92±0.05 | 9.00±0.09 | 9.10±0.10 | 9.17±0.06 | 9.11±0.01 | 9.17±0.07 | 9.19±0.04 | 9.22±0.06 | 9.24±0.04 | 9.28±0.06 |
| *Cellular community prokaryotes* | 2.18±0.03 | 2.15±0.01 | 2.13±0.01 | 2.15±0.02 | 2.14±0.01 | 2.14±0.02 | 2.15±0.01 | 2.17±0.02 | 2.19±0.02 | 2.21±0.01 | 2.20±0.00 | 2.20±0.02 | 2.21±0.01 | 2.21±0.01 | 2.19±0.01 | 2.21±0.03 |

Table S4. Relative abundance of significantly changed KEGG pathways at KEGG level 3 in plots that were monocropped with peanuts for different periods. Italic type represents the pathways with increased abundance and standard type represents pathways with decreased abundance.

|  | Y1.H20 | Y1.H26 | Y1.H50 | Y1.H917 | Y2.H20 | Y2.H26 | Y2.H50 | Y2.H917 | Y11.H20 | Y11.H26 | Y11.H50 | Y11.H917 | Y12.H20 | Y12.H26 | Y12.H50 | Y12.H917 |
| --- | --- | --- | --- | --- | --- | --- | --- | --- | --- | --- | --- | --- | --- | --- | --- | --- |
| DNA repair and recombination proteins | 2.78±0.01 | 2.79±0.01 | 2.80±0.01 | 2.81±0.01 | 2.76±0.04 | 2.77±0.03 | 2.78±0.01 | 2.77±0.00 | 2.68±0.01 | 2.68±0.01 | 2.72±0.01 | 2.74±0.01 | 2.72±0.00 | 2.74±0.01 | 2.76±0.01 | 2.74±0.02 |
| Purine metabolism | 1.84±0.00 | 1.84±0.00 | 1.85±0.01 | 1.86±0.01 | 1.85±0.03 | 1.83±0.02 | 1.84±0.00 | 1.84±0.00 | 1.79±0.01 | 1.77±0.03 | 1.79±0.02 | 1.81±0.01 | 1.81±0.01 | 1.81±0.02 | 1.82±0.01 | 1.82±0.03 |
| Transfer RNA biogenesis | 1.76±0.01 | 1.77±0.01 | 1.78±0.01 | 1.81±0.00 | 1.76±0.02 | 1.74±0.03 | 1.75±0.01 | 1.76±0.00 | 1.65±0.01 | 1.64±0.00 | 1.68±0.00 | 1.72±0.01 | 1.71±0.00 | 1.71±0.01 | 1.73±0.00 | 1.73±0.01 |
| Exosome | 1.54±0.00 | 1.55±0.00 | 1.55±0.00 | 1.55±0.01 | 1.56±0.02 | 1.55±0.01 | 1.55±0.00 | 1.55±0.00 | 1.53±0.01 | 1.52±0.02 | 1.53±0.01 | 1.539±0.01 | 1.53±0.00 | 1.53±0.02 | 1.53±0.01 | 1.53±0.02 |
| Amino acid related enzymes | 1.39±0.01 | 1.40±0.01 | 1.40±0.01 | 1.42±0.01 | 1.41±0.03 | 1.38±0.02 | 1.39±0.00 | 1.40±0.00 | 1.34±0.00 | 1.33±0.01 | 1.36±0.01 | 1.38±0.01 | 1.37±0.00 | 1.37±0.01 | 1.384±0.00 | 1.38±0.02 |
| Pyrimidine metabolism | 1.25±0.00 | 1.26±0.00 | 1.26±0.00 | 1.28±0.01 | 1.27±0.02 | 1.25±0.01 | 1.25±0.00 | 1.26±0.00 | 1.23±0.01 | 1.21±0.01 | 1.23±0.00 | 1.25±0.00 | 1.24±0.00 | 1.24±0.00 | 1.25±0.00 | 1.26±0.01 |
| Mitochondrial biogenesis | 1.22±0.00 | 1.22±0.00 | 1.22±0.00 | 1.22±0.00 | 1.23±0.00 | 1.23±0.00 | 1.22±0.00 | 1.23±0.01 | 1.18±0.02 | 1.19±0.01 | 1.20±0.01 | 1.20±0.01 | 1.20±0.01 | 1.20±0.01 | 1.20±0.00 | 1.20±0.02 |
| Ribosome | 1.17±0.01 | 1.18±0.00 | 1.19±0.00 | 1.20±0.01 | 1.21±0.04 | 1.18±0.01 | 1.18±0.01 | 1.19±0.00 | 1.16±0.01 | 1.141±0.02 | 1.16±0.01 | 1.17±0.01 | 1.17±0.00 | 1.16±0.01 | 1.17±0.01 | 1.17±0.02 |
| Oxidative phosphorylation | 1.16±0.01 | 1.17±0.01 | 1.18±0.01 | 1.19±0.00 | 1.17±0.01 | 1.15±0.02 | 1.16±0.00 | 1.16±0.00 | 1.10±0.01 | 1.10±0.01 | 1.12±0.00 | 1.14±0.01 | 1.13±0.00 | 1.13±0.01 | 1.14±0.00 | 1.14±0.01 |
| Carbon fixation pathways in prokaryotes | 1.08±0.01 | 1.09±0.00 | 1.09±0.00 | 1.09±0.01 | 1.11±0.01 | 1.10±0.01 | 1.09±0.00 | 1.09±0.00 | 1.07±0.01 | 1.07±0.01 | 1.07±0.01 | 1.07±0.01 | 1.07±0.00 | 1.06±0.01 | 1.06±0.01 | 1.07±0.02 |
| Aminoacyl tRNA biosynthesis | 1.08±0.01 | 1.08±0.00 | 1.09±0.01 | 1.10±0.01 | 1.10±0.03 | 1.08±0.01 | 1.08±0.00 | 1.08±0.00 | 1.04±0.01 | 1.03±0.00 | 1.05±0.00 | 1.06±0.00 | 1.06±0.00 | 1.06±0.01 | 1.06±0.00 | 1.07±0.00 |
| Alanine, aspartate and glutamate metabolism | 1.05±0.01 | 1.05±0.00 | 1.06±0.00 | 1.06±0.00 | 1.06±0.01 | 1.05±0.01 | 1.05±0.00 | 1.05±0.01 | 1.03±0.04 | 1.03±0.05 | 1.04±0.03 | 1.04±0.03 | 1.04±0.03 | 1.03±0.03 | 1.04±0.04 | 1.04±0.07 |
| *Ribosome biogenesis* | 1.54±0.01 | 1.53±0.02 | 1.51±0.03 | 1.48±0.02 | 1.59±0.04 | 1.60±0.06 | 1.58±0.02 | 1.59±0.02 | 1.70±0.05 | 1.73±0.05 | 1.70±0.03 | 1.63±0.03 | 1.65±0.03 | 1.65±0.03 | 1.58±0.04 | 1.60±0.08 |
| *Messenger RNA Biogenesis* | 1.42±0.02 | 1.42±0.03 | 1.40±0.04 | 1.35±0.02 | 1.50±0.05 | 1.50±0.07 | 1.47±0.02 | 1.48±0.02 | 1.61±0.04 | 1.64±0.05 | 1.60±0.03 | 1.52±0.03 | 1.55±0.03 | 1.53±0.03 | 1.46±0.05 | 1.47±0.08 |
| *RNA degradation* | 1.39±0.02 | 1.39±0.03 | 1.36±0.04 | 1.32±0.02 | 1.46±0.04 | 1.47±0.07 | 1.44±0.02 | 1.45±0.02 | 1.58±0.02 | 1.62±0.03 | 1.58±0.01 | 1.50±0.01 | 1.52±0.01 | 1.51±0.02 | 1.43±0.02 | 1.45±0.04 |
| *Quorum sensing* | 1.44±0.01 | 1.42±0.01 | 1.40±0.02 | 1.40±0.02 | 1.45±0.02 | 1.44±0.04 | 1.44±0.01 | 1.46±0.00 | 1.52±0.01 | 1.53±0.01 | 1.51±0.01 | 1.48±0.00 | 1.50±0.01 | 1.48±0.00 | 1.46±0.00 | 1.48±0.01 |
| *Glyoxylate and dicarboxylate metabolism* | 1.13±0.01 | 1.13±0.00 | 1.12±0.01 | 1.12±0.01 | 1.14±0.01 | 1.14±0.01 | 1.13±0.00 | 1.13±0.00 | 1.17±0.04 | 1.16±0.03 | 1.14±0.03 | 1.14±0.01 | 1.14±0.02 | 1.14±0.02 | 1.13±0.02 | 1.13±0.02 |
| *Lipid biosynthesis proteins* | 1.06±0.001 | 1.07±0.01 | 1.07±0.01 | 1.03±0.02 | 1.10±0.01 | 1.11±0.03 | 1.09±0.01 | 1.08±0.00 | 1.21±0.04 | 1.20±0.03 | 1.12±0.03 | 1.11±0.01 | 1.13±0.02 | 1.12±0.02 | 1.10±0.02 | 1.09±0.02 |
